# Cell Division Cycle 6 Promotes Mitotic Slippage and Contributes to Drug Resistance in Paclitaxel-Treated Cancer Cells

**DOI:** 10.1371/journal.pone.0162633

**Published:** 2016-09-09

**Authors:** Yue He, Daoyu Yan, Dianpeng Zheng, Zhiming Hu, Hongwei Li, Jinlong Li

**Affiliations:** Institute of Biotherapy, School of Biotechnology, Southern Medical University, Guangzhou, Guangdong, China; University of Hawaii System, UNITED STATES

## Abstract

Paclitaxel (PTX) is an antimitotic drug that possesses potent anticancer activity, but its therapeutic potential in the clinic has been hindered by drug resistance. Here, we report a mechanism by which cancer cells can exit from the PTX-induced mitotic arrest, i.e. mitotic slippage, and avoid subsequent death resulting in drug resistance. In cells experiencing mitotic slippage, Cdc6 protein level was significantly upregulated, Cdk1 activity was inhibited, and Cohesin/Rad21 was cleaved as a result. Cdc6 depletion by RNAi or Norcantharidin inhibited PTX-induced Cdc6 up-regulation, maintained Cdk1 activity, and repressed Cohesin/Rad21 cleavage. In all, this resulted in reduced mitotic slippage and reversal of PTX resistance. Moreover, in synchronized cells, the role of Cdc6 in mitotic exit under PTX pressure was also confirmed. This study indicates that Cdc6 may promote mitotic slippage by inactivation of Cdk1. Targeting of Cdc6 may serve as a promising strategy for enhancing the anticancer activity of PTX.

## Introduction

Microtubule has been a major target for the anticancer drugs development. The great success of PTX made it as an ‘epoch-making’ anticancer drug. PTX is currently one of the most widely used drugs for variously cancer chemotherapy [1]. Although PTX possess potent anticancer activity, it has been shown that treatment with this drug often results in resistance as well as undesirable side effects. Acquired resistance to this drug has become one of the major therapeutic obstacles. Therefore, mechanism clarification and hence possible strategies to overcome PTX resistance holds significant purpose [2].

PTX is a microtubule-stabilizing agent. It kills cells mainly by preventing microtubule depolymerization, triggering the spindle assembly checkpoint (SAC) to block cell cycle progression, and eventually results in cell apoptosis [3, 4]. However, cancer cells can resist such killing by premature exit from mitosis before cells initiate apoptosis either due to a weak checkpoint or rapid slippage [5]. The length of the arrested M phase is important for the cell fate. Prolonged M phase arrest allows the gradual accumulation of internal death signals in the cell [6]. However, increased slippage cause insensitivity to PTX-induced apoptosis [7]. Thus, blocking mitotic exit may be a better cancer therapeutic strategy for overcoming PTX resistance.

Cdc6 is a key component of the pre-replication complex (pre-RC) in initiating DNA replication in the G1 phase [8]. Recent studies demonstrated that, despite the licensing function for DNA replication, Cdc6 also regulates mitotic exit in from yeast to human cells [9]. Exit from mitosis requires the inactivation of mitotic Cdk1. In yeast, Cdc6 interacts with Cdk1 and contributes to Cdk1 inactivation in late mitosis. Deletion of Cdc6 lacking the Cdk-interacting domain has no effect on DNA replication duringS phase, but instead cause a delay in mitotic exit [10]. In human cells, interaction of Cdc6 with Cdk1 leads to Cdk1 inhibition and mitotic exit [11]. Thus, Cdc6 is clearly involved in Cdk1 inactivation during mitosis exit. In addition, Cdc6 is up-regulated in many types of cancer and is correlated with tumor malignant progression [12–14]. Deregulation of Cdc6 expression in human cells poses a serious risk of carcinogenesis [15]. However, the role of Cdc6 in premature mitotic exit under mitotic pressure is still poorly understood.

Norcantharidin (NCTD), a demethylated form of cantharidin, has profound anticancer activity against many kinds of cancer cells, including hepatocellular carcinoma [16], prostate cancer [17], and bladder cancer [18] et al. Previously researches demonstrated that NCTD induces degradation of the Cdc6 protein in cancer cells [19, 20] and Xenopus cell-free extracts system [9]. In this paper, mitotic slippage related to Cdc6 and drug resistance under PTX treatment was examined. The possible anti-mitotic slippage effect of NCTD or Cdc6 depletion in PTX-treated cells was explored. We are first to report that Cdc6 contributes to PTX-induced mitotic slippage and, more importantly, NCTD or Cdc6 RNAi inhibits the slippage and hence reverse the PTX resistance in cancer cells.

## Materials and Methods

### Cell culture and treatment

HepG2 and Hela cells were purchased from the ATCC and maintained in our lab. Cells were cultured in DMEM supplemented with 10% FBS, at 37°C under 5% CO_2_. PTX and Norcantharidin were purchased from Sigma-Aldrich. For Giemsa staining, cells were gently washed with phosphate-buffered saline (PBS) and fixed with cold methanol for 10 min. Then the cells were stained with Giemsa dye for 30 min and then examined by microscopy. The images were analyzed by Image-Pro Plus (version 6.0) software and the percentage of polyploid cells was calculated. For Typan Blue assay, cells were collected and washed by PBS and stained with Typan Blue dye for 1 min and then counted under microscopy.

### Cell cycle synchronization

Cells were synchronized at G1/S border by double thymidine block [17]. Briefly, cells were treated with 2 mM thymidine (purchased from Sigma-Aldrich) for 16 h, released into fresh medium containing 10% FBS for 9 h, then synchronized for a further 16 h with 2 mM thymidine. After washing three times with PBS, the block cells was released into fresh medium containing 10% FBS. Cells were harvested at 9, 13, 17 and 21 h. The cell-cycle progression was detected by flow cytometric analysis.

### Flow cytometry

Cells were fixed with 70% cold ethanol overnight at 4°C. Fixed cells were then washed once in ice-cold PBS and stained with propidium iodide (PI) staining solution (50μg/ml PI, 100μg/ml RNase, 0.05% Triton X-100 in PBS) for 30 min at 4°C in dark. PI-stained cells were then analyzed for their DNA content by flow cytometry (BD Biosciences, San Jose, CA, USA).

### Immunofluorescence

Mitotic cells were examined by pH3 immunostaining. Briefly, cells were fixed for 15 min in 4% (w/v) para-formaldehyde (PFA)/PBS and then permeabilized for 15 min in 0.25% (v/v) Triton X-100/PBS. Then cells were washed three times in PBS and then blocked with goat serum for 1 h. Cells were incubated with antibody against p-histone (pSer28) (KEYGEN) for 1 h, followed by incubation with fluorochrome-conjugated secondary antibody and DAPI (KEYGEN). Fluorescence images were taken under an Olympus fluorescent microscope. Image-Pro Plus (version 6.0) was used to analyse the fluorescent images. The mitotic index was calculated as pH3 cells/DAPI cells ×100%.

### SiRNA knockdown

SiRNA was used to deplete Cdc6 protein. Cells (5×10^5^) were seeded into 6-well plates and then were transfected with Cdc6-targeting siRNA (5-‘CCAAGAAGGAGCACAAGAUdTdT-3’) (Guangzhou Ribobio tech) in diluted Lipofectamine containing Opti-MEM Medium (Invitrogen) according to manufacturer’s protocol.

### Western blotting

Cells were lysed by ice-cold lysis buffer (50 mM Tris-HCl pH7.5, 150 mM NaCl, 1% NP40, 1 mM PMSF, and 10 units/ml aprotinin) supplemented with protease inhibitor cocktail tablets, for 20 min at 4°C, then centrifuged at 12,000 rpm for 10 min at 4°C. Proteins were separated by SDS-PAGE with 10% polyacrylamide gel. Membranes were incubated with anti-cdc6 antibody (Abcam, ab109315); anti-cdk1 antibody (Abcam, ab32384); anti-phospho-cdk1-Y15 antibody (Abcan, ab133463) or anti-RAD21 antibody (CST, D5Y8S). After washing with TBST, the membranes were incubated with HRP-conjugated secondary antibody at room temperature for 1 h. Signal detection was carried out with an ECL system (millipore, Billerica, MA, USA).

### Statistical analysis

Average values were expressed as mean ± standard deviation (S.D.). SPSS version 13.0 for Windows was used for all statistical analyses. Statistical comparisons were made using one-way ANOVA. *P* values of < 0.05 P were considered to be statistically significant.

## Results

### Mitotic slippage contributes to drug resistance in PTX-treated HepG2 cells

**Fig 1:**
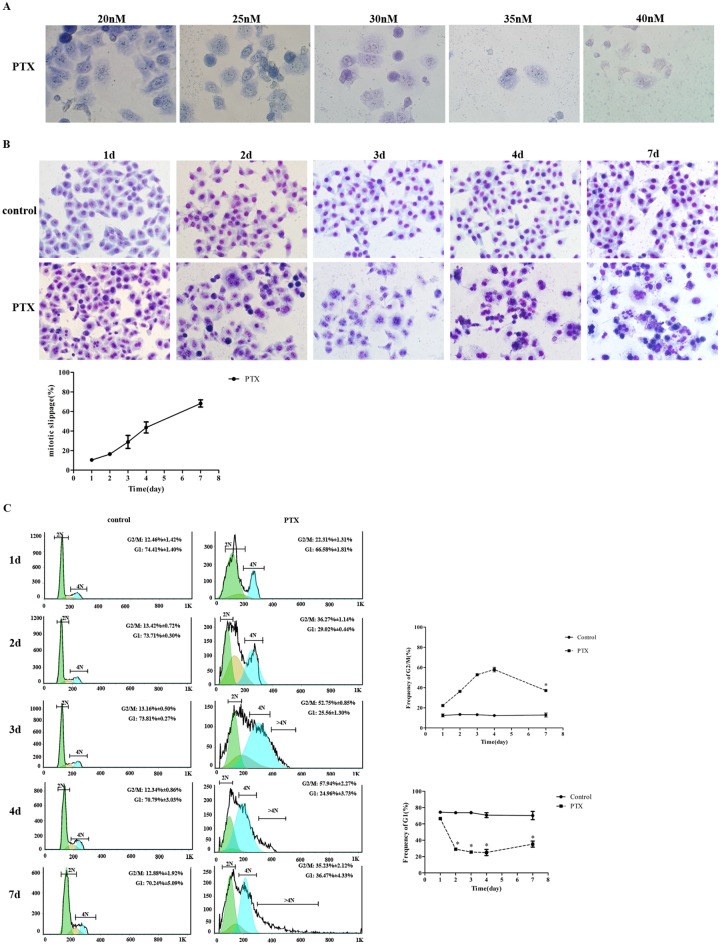
Mitotic slippage arises after PTX treatment. (A) HepG2 cell were treated with indicative concentration of PTX for 3 days or (B) with 30 nM PTX for 1 to 7 days. Cells were stained with Giemsa dye and viewed under bright field microscope. Representative photomicrographs are shown. The percentage of slippage cells is presented in below panel; (C) Cell cycle progression of (B) was analyzed by FACS; percentage of G2/M (upper) and G1 (below) are presented respectively in right panel. All of the experiments were performed in three independent experiments, *P<0.05.

Cancer cells have two major fates following the PTX-triggered mitotic arrest, they either die in mitosis through the activation of the intrinsic mitochondrial apoptosis pathway or escape from mitotic arrest in a process termed mitotic adaptation or slippage [21, 22]. In order to explore the relationship between slippage and resistance, Giemsa staining and FACS cell cycle analysis were performed to examine the mitotic progression and corresponding cell viability in PTX-treated cells. Our results show that in HepG2 cells, PTX caused mitotic arrest and killed cells in a dose- and time-dependent manner. However, in lower dose (30 nM) of PTX group, mitotic slippage cells, as demonstrated by DNA decondensation, polyploid cells in Giemsa staining (Fig 1A and 1B) and >4N DNA content in FACS analysis (Fig 1C), were seen after 2 days and became obvious (more than 50%) after 4 days PTX treatment. After PTX treatment for 7 days, polyploidy cells became the predominant populations (Fig 1B) and G2/M cells were significantly reduced (Fig 1C). At the same time, 2N cells were significantly increased (Fig 1C, right panel). These results indicate that resistance to PTX is related to increased mitotic slippage. Our results are consistent with previous research, which have shown that polyploid cells is basis for chemo-resistance and stem-like properties [23, 24].

### Cdc6 contributes to PTX-induced mitotic slippage by inactivation of Cdk1

**Fig 2:**
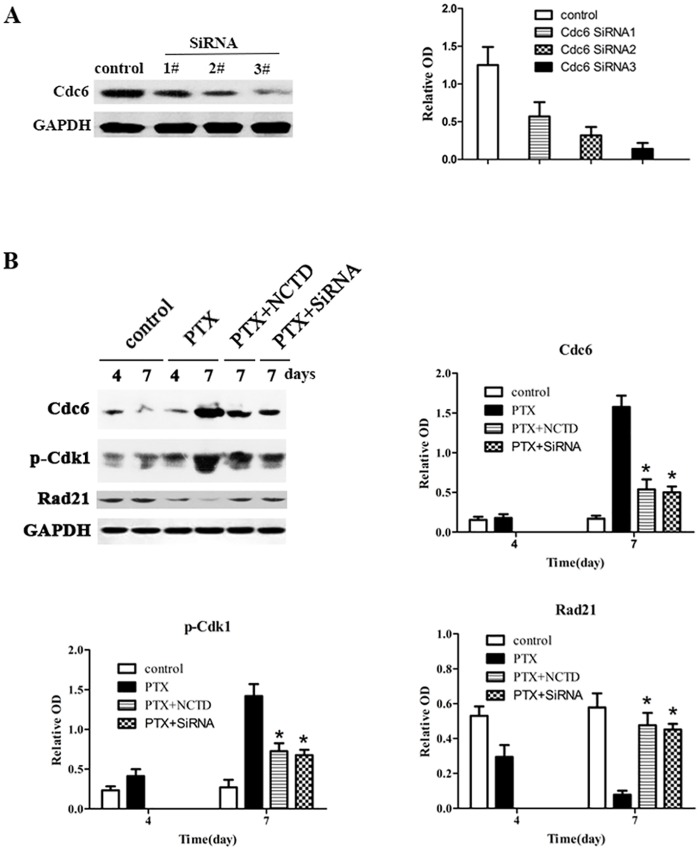
Cdc6 contributes to PTX-induced mitotic slippage by inactivation of Cdk1. (A) HepG2 cells were transfected with siRNA targeting cdc6 mRNA for 24h, the inhibitory effect was examined by Western Blotting. (B) HepG2 cells were treated with PTX (30 nM) for 4 and 7 days, NCTD (30 μM) was added to replace PTX after 4 days treatment. Cdc6 RNAi was transfected after PTX treatment for 4 days. Cdc6, pCdk1 and Rad 21 were examined by Western Blotting. GAPDH was used as loading control. The protein levels are expressed as optical density fold difference related to GAPDH (relative OD). Three independent experiments were performed, *P<0.05 as compared to PTX group.

To explore the role of Cdc6 on PTX-induced mitotic slippage, we examined the protein levels of Cdc6 and phosphorylated Cdk1 after PTX treatment. The latter is a conserved tyrosine phosphorylation (Tyr15 in humans) leading to inhibition of Cdk1 activity [25]. To deplete Cdc6, NCTD administration or RNAi transfection was performed after 4 days PTX treatment when considerable slippage cells emerged. Western Blotting showed that 3# siRNA efficiently inhibited the Cdc6 expression (Fig 2A). Our results show that PTX significantly up-regulated Cdc6 protein level after 7 days treatment. The p-Cdk1 was maintained at a high level, indicating that Cdk1 activity was inhibited. Separase was also activated, as demonstrated by cleavage of Rad21. NCTD or Cdc6 RNAi down-regulated Cdc6 protein level and, more importantly, reduced p-Cdk1 level and inhibited the cleavage of Rad21 (Fig 2B). These results demonstrate that Cdc6 contributes to mitotic slippage by inactivating of Cdk1.

### Cdc6 depletion inhibits PTX-induced mitotic slippage and reverses PTX resistance

**Fig 3:**
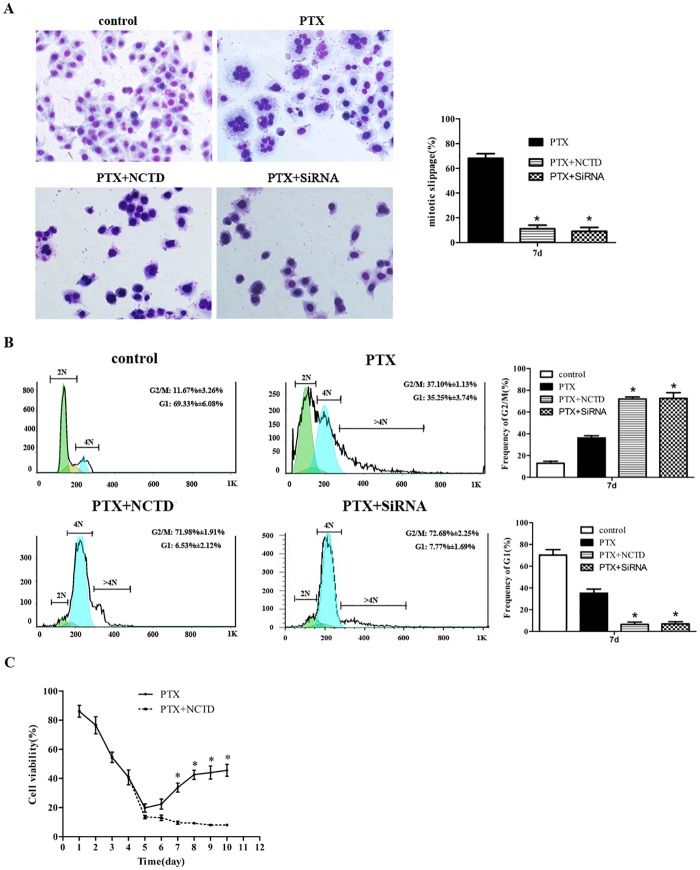
Cdc6 depletion inhibits PTX-induced mitotic slippage and reverses PTX resistance. (A) HepG2 cells were treated with combination with or without Cdc6 RNAi or NCTD (30 μM) as described in Fig 2. At day 7, Cells were stained with Giemsa dye and the percentage of slippage cells is presented (right panel). (B) Cell cycle was analyzed by FACS. G2/M (upper) and G1 (below) percentage were presented in right panel. (C) Cell viability after PTX treatment combined with or without NCTD (30 μM) for 10 days was valued by typan blue assay. All of the experiments were performed in three independent experiments, *P<0.05 as compared to PTX group.

To test the Cdc6 depletion effect on mitotic slippage and cell viability, RNAi or NCTD were used as described above to deplete Cdc6. NCTD or Cdc6 RNAi successfully inhibited appearance of polyploidy cells (Fig 3A) and arrested cells in mitosis after PTX treatment for 7 days (Fig 3A and 3B). More importantly, NCTD inhibited the increase of cell viability after 4 days PTX treatment (Fig 3C).

### Cdc6 depletion inhibits mitotic slippage in synchronized cells

**Fig 4:**
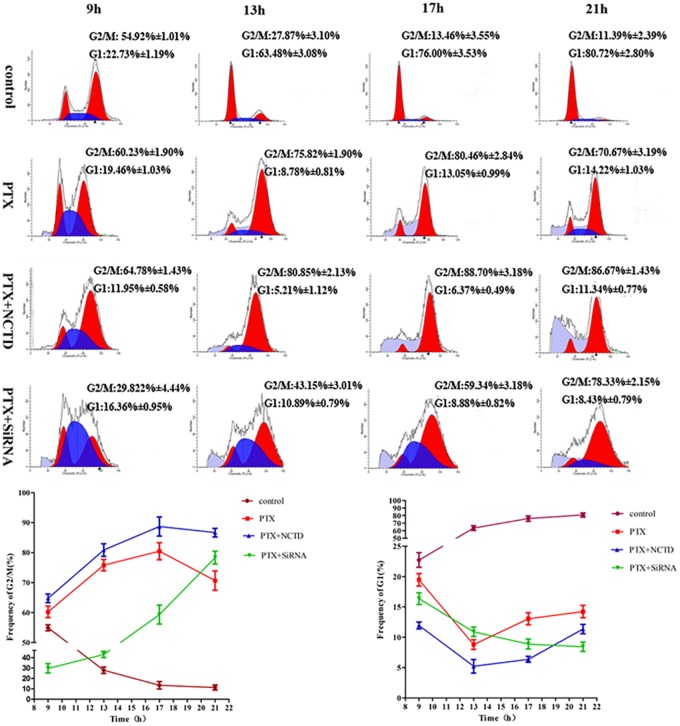
Cdc6 depletion inhibits mitotic exit in synchronized cells. HepG2 cells were synchronized by double thymidine block, and then released into fresh medium containing PTX (30 nM) or PTX (30nM) + NCTD (30 μM). For Cdc6 RNAi, the transfection was performed after first thymidine block. Cell cycle profiles were analyzed by FACS after released for indicative time. G2/M (left) and G1 (right) percentage are presented in below panel. Three independent experiments were performed.

To further characterize the Cdc6 depletion effect on mitotic slippage, we analyzed the cell cycle progression in late G1 synchronized cells. In the control group, after release from double thymidine block for 9h, cells progressed to M phase; after release for 13h, most cells went through mitosis and progressed into G1 phase. In the PTX group, cells went through S normally and accumulated in G2/M phase (Fig 4). However, the G1 fraction gradually increased from 13 to 21h and the G2/M cells began to decline at 21 h (Fig 4 below panel), indicating that a fraction of cells slipped out of mitosis and progressed into G1. It should be noticed that the mitotic slippage occurred promptly, as early as 17h after release in our experiments, as demonstrated by the increased G1 cells at 17 h compared to 13 h. For NCTD combination treatment, cells were blocked in G2/M phase, with much lower percentage of cells progressing into next G1 phase when compared to PTX treatment (Fig 4 below panel). Interestingly, obvious subG1 accumulation was observed at 21h, indicating cell apoptosis was induced (Fig 4). Cdc6 RNAi however showed different profiles on cell cycle progression. After release, the S phase progression is slower as compared with other groups (Fig 4), indicating the DNA replication was retarded. The G2/M phase cells were gradually increased and the mitotic slipped G1 cells were also inhibited (Fig 4 below panel).

**Fig 5:**
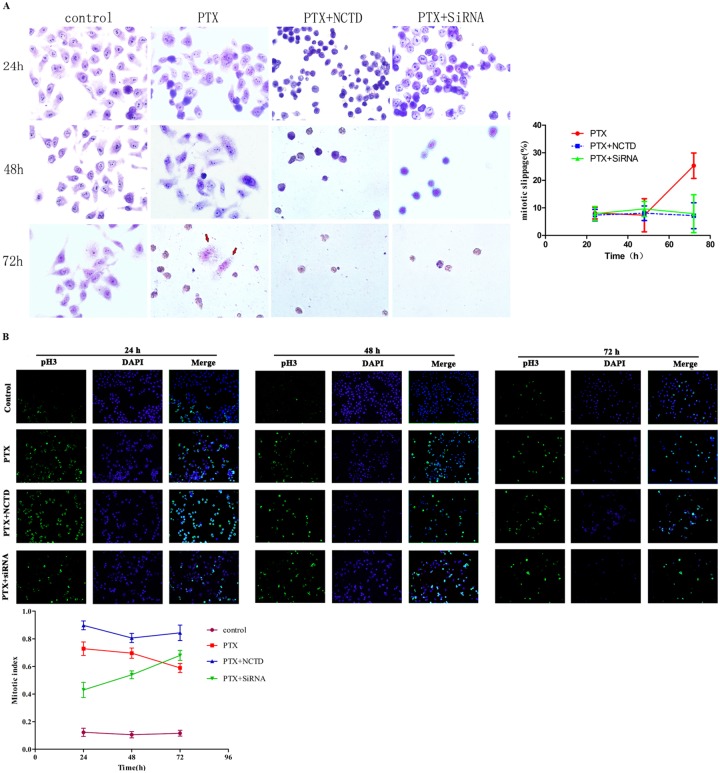
Cdc6 depletion inhibits mitotic slippage in synchronized cells. The synchronized cells in Fig 4 were collected after released for 24, 48 and 72h. (A) Cells were stained with Giemsa dye and the percentage of slippage cells is presented in (right panel). (B) Mitotic cells were examined by pH3 immunostaining and the mitotic index was calculated as pH3 cells/DAPI cells ×100% (below panel).

Next, we examined Cdc6 depletion effect on mitotic slippage in the synchronized cells after release for 24, 48, and 72h. Cdc6 depletion kept cells arrested in mitosis and caused more severe cell death (Fig 5A). The pH3 (a mitotic cell marker) immunostaining showed a decreased mitotic index trend after PTX treatment, indicating mitotic slippage happened. Higher mitotic index were obtained with Cdc6 RNAi or NCTD combination after 72 h (Fig 5B). It is interesting that in PTX+Cdc6 RNAi group, the mitotic index at 12 h is lower than PTX alone, but it kept increasing from 24 to 72 h, and reached a higher level than PTX treatment alone (Fig 5B1). The initial lower mitotic index may attribute to the efficient inhibition of DNA replication by Cdc6 depletion, which would slow down the S progression.

**Fig 6:**
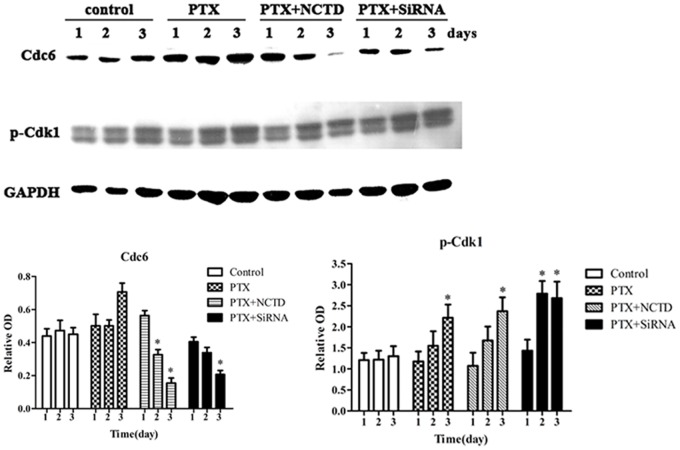
Cdc6 contributes to PTX-induced mitotic slippage in synchronized cells. The synchronized cells in Fig 5 were collected to extract total proteins. Cdc6 and pCdk1 were examined by Western Blotting. The protein levels are expressed as relative OD. All of the experiments were performed in three independent experiments, *P<0.05 as compared to PTX group.

Western Blotting shows that PTX caused Cdc6 up-regulation and Cdk1 inactivation. NCTD or Cdc6 RNAi reversed the PTX-induced Cdc6 up-regulation, but leaving Cdk1 activation no significant change (Fig 6). We deduced that may be due to the relative low percentage of slippage cells after 3 days PTX treatment.

### Cdc6 depletion inhibits mitotic slippage in Hela cells

**Fig 7:**
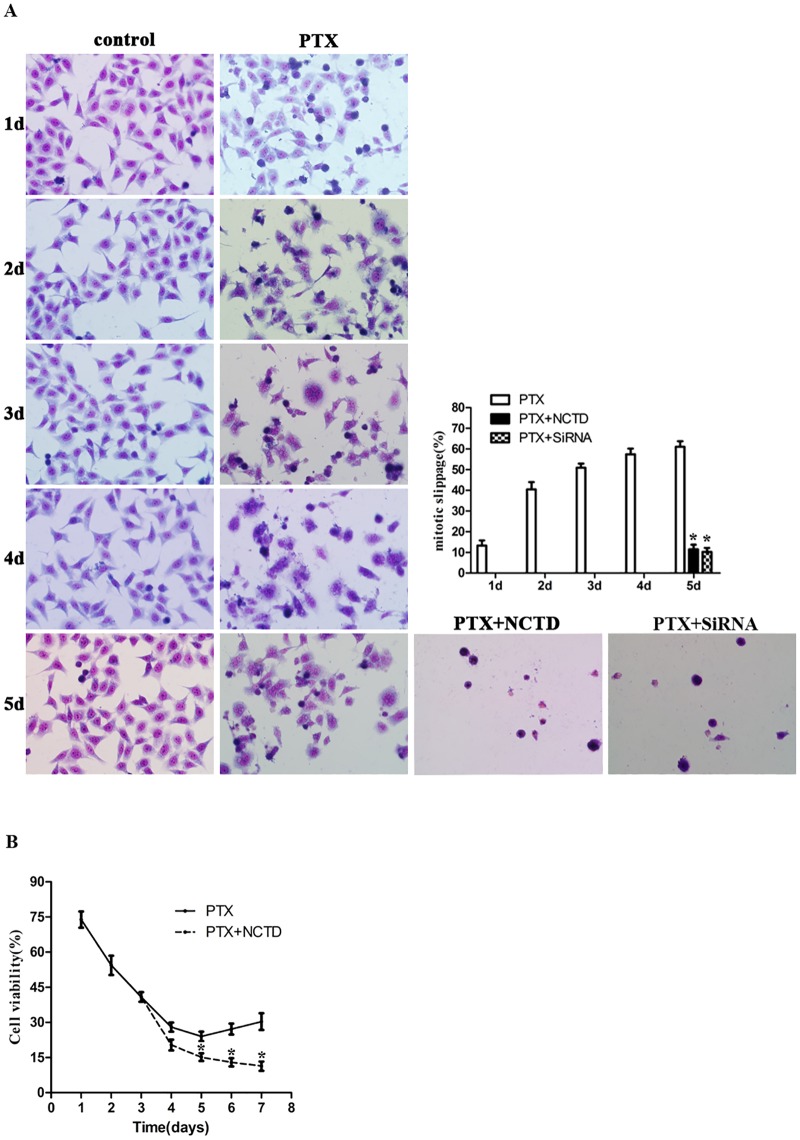
Cdc6 depletion inhibits PTX-induced mitotic slippage in Hela cells. (A) Hela cells were treated with PTX (30 nM) for 1 to 5 days, NCTD (30 μM) was added to replace PTX after 3 days PTX treatment. Cdc6 RNAi was transfected after PTX treatment for 3 days. Cells were stained with Giemsa dye and the percentage of slippage cells is calculated (right panel). (B) Cell viability after PTX treatment combined with or without NCTD (30 μM) for 7 days was valued by typan blue assay. All of the experiments were performed in three independent experiments, *P<0.05 as compared to PTX group.

In order to exclude the cell type specific events for Cdc6’ effect on mitotic slippage, we examined the anti-slippage effect of NCTD or Cdc6 RNAi on Hela cells. Our results show that, similar to HepG2 cells, Hela cells underwent mitotic slippage after PTX treatment. The slippage occurred more readily and earlier than HepG2 cells, which became obvious after 3 days PTX treatment (Fig 7A). NCTD or Cdc6 RNAi efficiently inhibited the mitotic slippage in Hela cell (Fig 7A). Furthermore, cell viability increased gradually from day 5 after PTX treatment. NCTD efficiently inhibited the increased survival tendency (Fig 7B).

**Fig 8:**
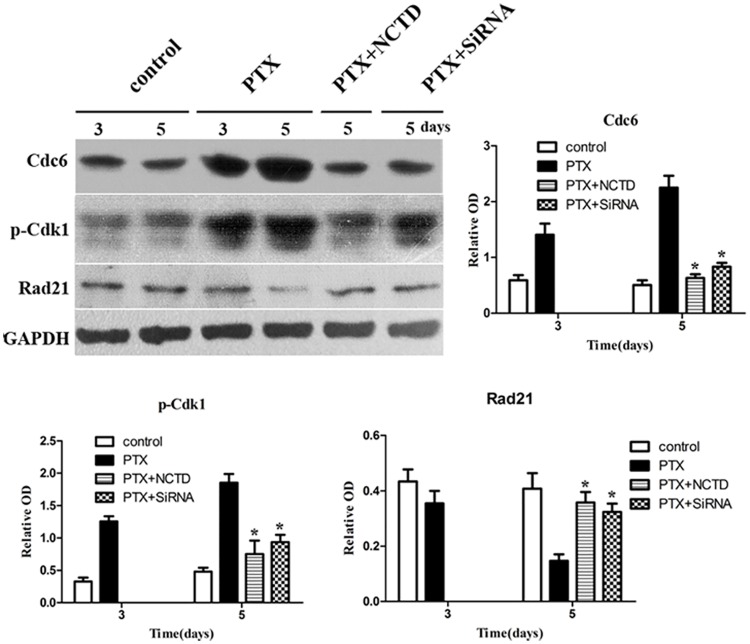
Cdc6 depletion reverses the PTX-induced Cdk1 inactivation in Hela cells. Hela cells were treated with PTX (30 nM) combination with or without Cdc6 RNAi or NCTD (30 μM) as described in Fig 7. Cdc6, pCdk1 and Rad 21 were examined by Western Blotting. GAPDH was used as loading control. The protein levels are expressed as optical density fold difference related to GAPDH (relative OD). Three independent experiments were performed, *P<0.05 as compared to PTX group.

WB results show that in Hela cells, PTX up-regulated Cdc6 protein level, maintained p-Cdk1 in high level, and induced Rad21 cleavage. NCTD or Cdc6 RNAi down-regulated Cdc6, reduced p-Cdk1 level and inhibited the cleavage of Rad21 (Fig 8). These results are similar to that in HepG2 cells, indicating that Cdc6 contributes to mitotic slippage in cancer cells.

## Discussion

Cancer cells can acquire resistance to PTX by ordinary means, i.e., MDR, point mutations, and overexpression of tubulin subtypes [1]. However, considering the unique cytotoxic mechanism of these microtubule-poisoning agents, the cell response to mitotic arrest is most important in determining the outcome of these drugs. We have shown here that mitotic slippage results in PTX resistance in cancer cells. Cdc6 depletion blocks the mitotic slippage and prolongs the mitotic arrest upon PTX treatment. More importantly, Cdc6 depletion reverses the PTX resistance on cancer cells. These results are consistent with previous research, which have shown that increased slippage contributed to the resistance of PTX-induced apoptosis in MDA-MB-231 cells [7]. Blocking mitotic exit by Cdc20 knockdown was shown to inhibit mitosis slippage and overcome apoptosis-resistant in cancer cells [5].

In this study, mitotic slippage was accompanied by up-regulation of Cdc6 after PTX treatment. Cdc6 depletion prevented the mitotic slippage and blocked cell in mitosis. Cdc6 is reported to act as a Cdk1 inhibitor to promote mitotic exit [10, 11]. The inactivation of Cdk1 is important for M phase cells to achieve chromosome segregation. Separase triggers anaphase by hydrolyzing its substrate cohesin, which is the protein that bind sister chromatids during the early stage of anaphase. Before anaphase, separase is inhibited by association with Cdk1. The inactivation of Cdk1 releases separase, triggers cohesin degradation, and initiates the final separation of sister chromatids [26]. In our research, after PTX treatment for 7 days, plenty of mitotic slippage cells arise. In these cells, Cdc6 protein level significantly increased and the Cdk1 activity was inhibited. Accordingly, Rad21 was cleaved. Cdc6 depletion down-regulated Cdc6 protein, reversed the inactivation of Cdk1 and kept most Rad21 in intact form. These results suggest that Cdc6 promotes mitotic slippage by inactivation Cdk1; NCTD may exert anti-mitotic slippage effect via degradation of Cdc6.

By limiting Cdk1 activity, Cdc6 not only promotes the M-phase exit, but also inhibits M-phase entry. Overexpression of Cdc6 in G2 phase cells prevents entry into mitosis in HeLa cells [27]. Addition of Cdc6 in Xenopus embryo cycling extract delays the mitotic entry. While down regulation of endogenous Cdc6 accelerates the mitotic entry and increases the level of Cdk1 activity during the M-phase [9]. Thus, it seems that Cdc6 acts as a regulator to avoid cell staying in mitosis, especially when cell is under replication stress and abnormal mitosis.

It should be noticed that most slipped cells is doomed to die. There are reports that mitotic slippage contributes to the extra cytotoxicity of PTX. RO3306 (Cdk1 inhibitor) enhance apoptosis in PTX treated cells by mitotic slippage [28]. Forced mitotic exit by physiological hyperthermia significantly increased PTX cytotoxicity [29]. It seems that promoting mitotic exit may be more powerful for PTX to kill cells than blocking mitotic exit. But, despite the fact that most of the cells that slipped out of PTX-imposed mitotic arrest die in the subsequent cell cycles, there will be cells survive, partly attributing to the survival advantage which acquired from its disordered genome [23, 24]. In addition, the post-slippage apoptosis is likely mediated by the p53-dependent DNA damage response pathway [30]. While in most tumors, p53 is negative and incapable of p53-dependent apoptosis. So, it is suggested that PTX directly affects cells only in mitosis, and the duration of mitosis determines cell fate, including p53-dependent G1-like arrest [31]. Accordingly, lower mitotic index and multinuclear cells are related to survival advantage in tumors exposed to PTX [32]. Recently, it has been reported that stress-induced (carboplatin or hypoxia) polyploid cells contributes to chemo-resistance and shows stem-like properties [23, 24]. In our research, polyploid cells were observed after PTX treatment, indicating that cells survived the toxin and went through several rounds of cell cycle. More importantly, these slippage polyploid cells are resistant to PTX. Preventing the mitotic slippage (by NCTD or Cdc6 RNAi) may keep the cells in mitosis and decrease the chance of cells to get survival mutation.

We show here that Cdc6 contributes to PTX resistance by promoting mitotic slippage. Cdc6 depletion could inhibit the mitotic slippage and reverses PTX resistance under PTX pressure. Therefore, blocking mitotic exit is an effective way to enhance killing effect of PTX. Cdc6 may be served as promising target for improving the therapeutic effect of microtubule-poisoning drugs in cancer treatment.
